# Comparison of Cellular Uptake and Inflammatory Response via Toll-Like Receptor 4 to Lipopolysaccharide and Titanium Dioxide Nanoparticles

**DOI:** 10.3390/ijms140713154

**Published:** 2013-06-26

**Authors:** Sharmy Saimon Mano, Koki Kanehira, Akiyoshi Taniguchi

**Affiliations:** 1 Cell-Materials Interaction Group, Biomaterials Unit, Nano-Life Field, International Center for Materials Nanoarchitectonics (MANA), National Institute for Materials Science, 1-1, Namiki, Tsukuba, Ibaraki 305-0044, Japan; E-Mail: saimon.sharmy@nims.go.jp; 2 Graduate School of Advanced Science and Engineering, Waseda University, 3-4-1 Okubo, Shinjuku, Tokyo 169-8555, Japan; 3 Biotechnology Group, TOTO Ltd. Research Institute, Honson 2-8-1, Chigasaki, Kanagawa 253-8577, Japan; E-Mail: koki.kanehira@jp.toto.com

**Keywords:** toll-like receptors, titanium dioxide nanoparticles, LPS binding protein, CD 14

## Abstract

The innate immune response is the earliest cellular response to infectious agents and mediates the interactions between microbes and cells. Toll-like receptors (TLRs) play an important role in these interactions. We have already shown that TLRs are involved with the uptake of titanium dioxide nanoparticles (TiO_2_ NPs) and promote inflammatory responses. In this paper, we compared role of cellular uptake and inflammatory response via TLR 4 to lipopolysaccharide (LPS) and TiO_2_ NPs. In the case of LPS, LPS binds to LPS binding protein (LBP) and CD 14, and then this complex binds to TLR 4. In the case of TiO_2_ NPs, the necessity of LBP and CD 14 to induce the inflammatory response and for uptake by cells was investigated using over-expression, antibody blocking, and siRNA knockdown experiments. Our results suggested that for cellular uptake of TiO_2_ NPs, TLR 4 did not form a complex with LBP and CD 14. In the TiO_2_ NP-mediated inflammatory response, TLR 4 acted as the signaling receptor without protein complex of LPS, LBP and CD 14. The results suggested that character of TiO_2_ NPs might be similar to the complex of LPS, LBP and CD 14. These results are important for development of safer nanomaterials.

## 1. Introduction

Nanomaterials are being applied in the biological fields of bio-imaging [1,2], biosensing [3], drug delivery [4,5], cancer targeting [6] and detection [7], diagnostics [8], and therapeutics [9]. However, there is a lack of information regarding the potential toxicity of those materials in cells and organisms. It is thus essential to evaluate the safety of such materials. Nanotoxicology [10], an emerging field, is the toxicological evaluation of nanomaterials both *in vivo* and *in vitro*. Different parameters can determine the cytotoxicity of nanomaterials. Among these, the size and composition of the nanomaterial, as well as the target cell types, are the critical factors [11].

Titanium dioxide (TiO_2_) has been used for many years in a vast range of industrial and consumer products such as paints, pigments, cosmetics, and skin care products. Personal care products, tooth paste and sunscreen contained 1% to >10% TiO_2_ by weight while other products contained the lowest level of TiO_2_ (<0.01 μg/mL) [12]. TiO_2_ nanoparticles (NPs) form aggregates within minutes [13]. The use of TiO_2_ in consumer products does not mean it is safe. The nano-sized TiO_2_ in sunscreens results in bioaccumulation and chronic toxicity [14]. Studies on TiO_2_ NPs have shown that it destabilizes the cell membranes of digestive gland tubes *ex vivo* [15]. Ultrafine TiO_2_ NPs induce oxidative stress and inflammatory responses in human lung epithelial cells [16]. However, functional modifications of TiO_2_ show biocompatibility [17]. When TiO_2_ is doped with Au and Pt is effective in killing cancer cells [18].

The innate immune system is designed to provide a rapid response to pathogens and is thus known as the first line of defense. Toll like receptors (TLRs) play a critical role in early innate immunity to invading foreign pathogens such as microorganisms [19]. These receptors recognize distinct pathogen-associated molecular patterns that are expressed on infectious agents. The activation of this receptor mobilizes nuclear factor kappa B (NF-κB), which in turn activates a host of inflammatory-related target genes. Genes encoding 10 TLRs (TLR 1 to TLR 10) have been identified in the human genome. Among these TLRs, TLR 1, 2, 4, 5, and 6 are present on the cell surface, and TLR 3, 7, 8, and 9 expressed intracellularly. We hypothesize that TLRs also play important roles in the interactions between NPs and cells. We have shown that TLRs are involved in the uptake of TiO_2_ NPs and promote the associated inflammatory responses [20–22]. In addition, other studies have shown that TLRs play a vital role in the interaction between cells and nanomaterials [23,24]. A recent study showed that TiO_2_ NPs stimulate inflammatory responses in mice followed by apoptosis and lung injury through the activation of TLR2 or TLR 4 [25]. However, there is little information concerning the specific interactions.

Lipopolysaccharide (LPS) [26] is a major component in the outer membrane of Gram-negative bacteria and is a well-known inducer of the innate immune response, and LPS is major ligand of TLR 4. Cellular activation by LPS requires a complex formation with lipopolysaccharide binding protein (LBP) and cluster of differentiation (CD 14) [27]. LBP [28] has the functional capacity to bind with LPS [29]. The LPS:LBP complex is subsequently delivered to CD 14, which is anchored on the membrane (mCD 14) via a glycosyl phosphatidylinositol (GPI) tail or in a soluble form (sCD 14) [30]. The LPS:LBP:CD14 complex then interacts with TLR 4 [31]. In the case of an LPS-induced immune response, it is necessary for LPS to form a complex with LBP and CD 14 for uptake as well as signal transduction. In the case of NP-mediated signal transduction, it has not yet been demonstrated that LBP and CD 14 are involved in the uptake and signaling.

Our goal was to understand how to interact TiO_2_ NPs and TLR 4. This information will be useful for development of safety nanomaterials. In this paper, in order to find out whether LBP and CD 14 take part in the uptake and inflammatory signaling of TiO_2_ NPs, we compared role of cellular uptake and inflammatory response via TLR 4 to LPS and TiO_2_ NPs. For cellular activation, TLR 4 does not form a complex with LBP and CD 14. The results suggested that character of TiO_2_ NPs might be similar to the complex of LPS, LBP and CD 14. Our findings are important for understanding of interaction between NPs and cells, which is essential in developing the biosafety of NPs.

## 2. Results and Discussion

### 2.1. TLR 4, but Not LBP or CD 14, Is Involved in the Inflammatory Signal Transduction Mediated by TiO_2_ NPs

We have already shown that TiO_2_ NPs induce inflammatory cytokines such as IL-6 in NCI-H292 cells [32,33]. We have also discovered that TLR 4 expression vector-transfected cells increase the uptake of TiO_2_ NPs and also increase IL-6 mRNA upon exposure to TiO_2_ NPs [20]. In this paper, we compared the role of cellular uptake and inflammatory response via TLR 4 to LPS and TiO_2_ NPs. In the case of LPS, LPS binds to LBP, and then a complex of LPS and LBP binds to CD 14. Then, the complex of LPS, LBP, and CD 14 binds to TLR 4. After that, the signaling induces the inflammatory response.

In the case of TiO_2_ NPs, the necessity of LBP and CD 14 to induce the inflammatory response was investigated. We conducted a series of experiments in which the cells were transfected with different human expression vectors such as LBP, CD 14, TLR 4, or co-transfections of two or three of these vectors such as LBP:TLR 4, CD 14:TLR 4, or LBP:CD 14:TLR 4. LPS was used as a positive control.

**Figure 1:**
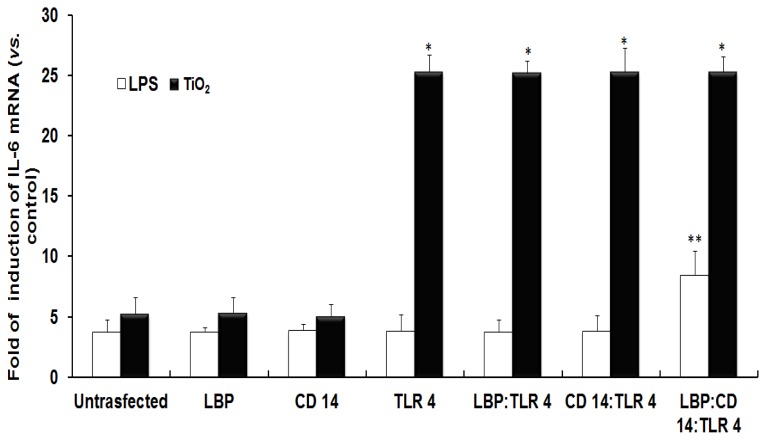
Induction of IL-6 mRNA in human expression vector-transfected NCI-H292 cells. NCI-H292 cells were transfected with or without human expression vectors such as lipopolysaccharide (LPS) binding protein (LBP), cluster of differentiation (CD) 14, toll-like receptor (TLR) 4, or were co-transfected. The expression of IL-6 mRNA was analyzed using quantitative real-time PCR. Each histogram shows the relative fold of induction of IL-6 mRNA in LPS (open bars) or TiO_2_ NPs (solid black bars) exposed NCI-H292 cells. The data were normalized against untreated cells. The significance was determined by comparison with un-transfected cells. Each bar represents mean ± SD, *n* ≥ 3 for each bar. * *p* ≤ 0.05, ** *p* < 0.005.

Figure 1 shows that the fold induction of IL-6 mRNA in NCI-H292 cells transfected with different expression vectors followed by exposure to LPS or TiO_2_ NPs. We first checked the expression level of IL-6 mRNA in NCI-H292 cells exposed to LPS or TiO_2_ NPs without the transfection of any of the expression vectors. The results show that LPS or TiO_2_ NPs induced IL-6 mRNA to 3.7 or 5.2 times respectively greater than that of the control cells that were not exposed to NPs (Figure 1, Untransfected). Next, we performed the same experiment in cells transfected with various expression vectors. In the case of LPS, the levels of IL-6 mRNA were approximately two-fold greater after transfection with LBP:CD14:TLR 4 compared with untransfected cells. This result indicated that LBP, CD 14, and TLR 4 are involved in the inflammatory signal transduction mediated by LPS (Figure 1, see white bars). In the case of TiO_2_ NP-exposed cells, the levels of IL-6 mRNA were approximately five-fold greater after transfection with TLR 4, LBP:TLR 4, CD 14:TLR 4, or LBP:CD 14:TLR 4 compared with untransfected cells; however, levels of IL-6 mRNA in LBP and CD 14 transfected cells were almost the same as those in untransfected cells (Figure 1, see black bars). These results indicated that IL-6 mRNA was induced by transfection of TLR 4, while transfection of LBP or CD 14 did not induce IL-6 mRNA expression in TiO_2_ NP-exposed cells. This result suggests that TLR 4 is involved in the inflammatory signal transduction mediated by TiO_2_ NPs.

**Figure 2:**
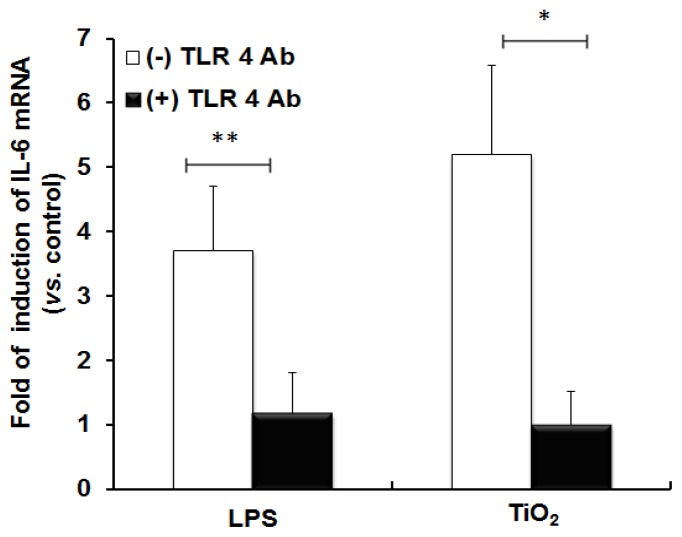
Inhibition of the expression of IL-6 mRNA in NCI-H292 cells incubated with anti-TLR 4 Ab. NCI-H292 cells were incubated with anti-TLR 4 Ab followed by exposure to LPS or TiO_2_ NPs. The expression of IL-6 mRNA was analyzed in anti-TLR 4 Ab-treated (solid black bars) and untreated (open bars) groups. The histograms show the variation in the induction of IL-6 mRNA. The data were statistically analyzed for the degree of significance. Each histogram represents mean ± SD, where *n* = 3 for each. * *p* < 0.05, ** *p* < 0.01.

In order to confirm the role of TLR 4 in inflammatory signal transduction, we blocked the function of TLR 4 by incubating the cells with anti-TLR 4 Ab followed by exposure to TiO_2_ NPs. Figure 2 shows the expression of IL-6 mRNA in anti-TLR 4 Ab-treated and un-treated cells. Anti-TLR 4 Ab treatment diminished the inflammatory response in cells, similar to LPS treatment. This data confirmed that TLR 4 is involved in the inflammatory signaling induced by TiO_2_ NPs.

**Figure 3:**
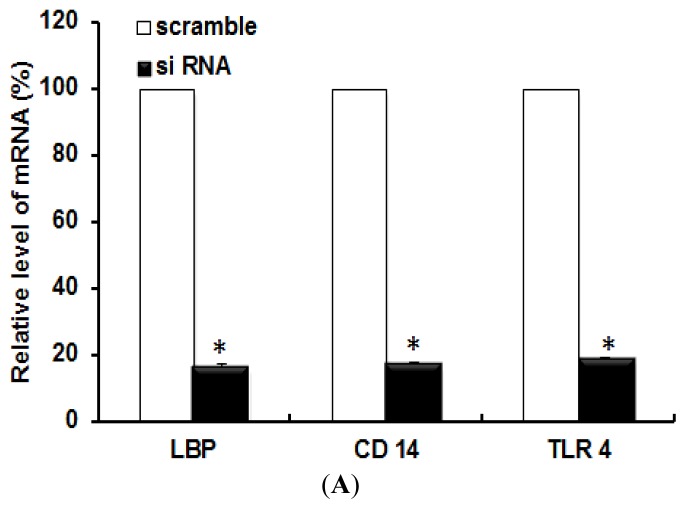

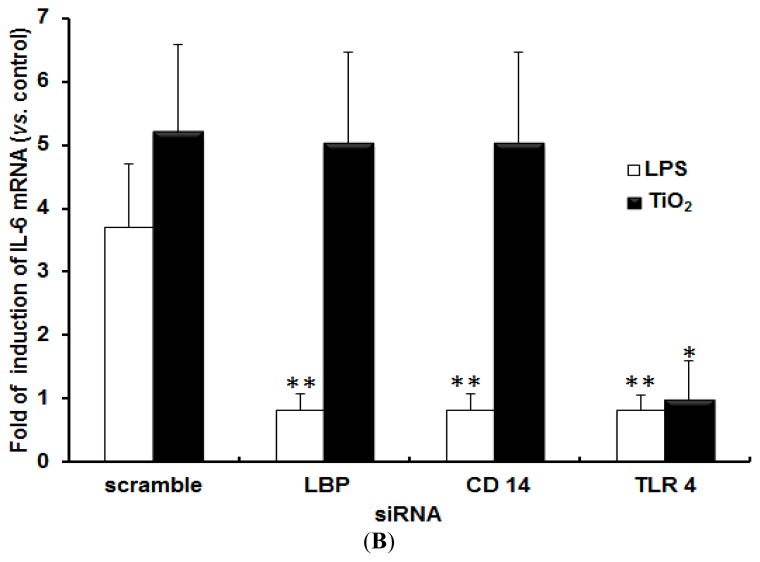
Gene silencing to knockdown specific target genes. (**A**) Gene silencing was confirmed in NCI-H292 cells transfected with scrambled siRNA (open bars) or siRNA specific for LBP, CD 14, or TLR 4 (solid black bars); (**B**) Cells were subjected to siRNA experiments followed by exposure to LPS (open bars) or TiO_2_ NPs (solid black bars) for 6 h. Each histogram represents IL-6 mRNA expression in siRNA-treated cells. Each graph was compared with that from the corresponding scrambled siRNA for statistical significance. Each bar represents mean ± SD, *n* = 3 for each. * *p* ≤ 0.05, ** *p* < 0.005.

Gene silencing was also performed to knockdown specific genes such as LBP, CD 14, or TLR 4. The knockdowns of the target genes were confirmed by the expression of those genes in siRNA transfected cells by RT-PCR (Figure 3A). A scrambled siRNA was used as a control for siRNA transfection experiments. After the gene knockdown, the cells were treated with LPS or TiO_2_ NPs for 6 h, and the induction of IL-6 mRNA was measured. Figure 3B shows the inflammatory response of gene knocked-down cells treated with LPS or TiO_2_ NPs. In the case of LPS treatment, gene knockdown of LBP, CD 14, or TLR 4 reduced the expression of IL-6 mRNA compared with the control (Figure 3B, white bars). In the case of TiO_2_ NP treatment, the results showed that the expression of IL-6 mRNA was reduced by TLR 4 knockdown, but not by LBP or CD 14 knockdown (Figure 3B, black bars). This result also confirmed that TLR 4, but not LBP or CD 14, takes part in the inflammatory signal transduction by TiO_2_ NPs.

### 2.2. TLR 4, but Not LBP or CD 14, Is Involved in the Uptake of TiO_2_ NPs to Cells

**Figure 4:**
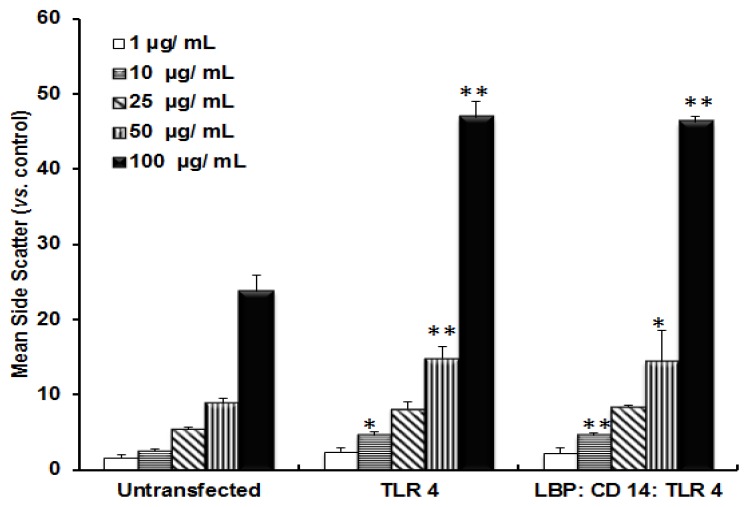
Incorporation of TiO_2_ NPs into NCI-H292 cells transfected with expression vectors. NCI-H292 cells were transfected with or without TLR 4 expression vector, or triple co-transfected with LBP, CD 14, and TLR 4 vectors. The side scatter was analyzed by flow cytometry and the mean side scatter for TiO_2_ NP-exposed cells was calculated from control cells (without NPs). The degree of significance for each concentration (1 μg/mL to 100 μg/mL) was calculated from the corresponding concentration of NP-exposed un-transfected cells. Each bar represents each concentration of NPs. The data represent means ± SD, *n* = 3 for each concentration. * *p* ≤ 0.02, ** *p* ≤ 0.005.

The incorporation of TiO_2_ NPs by cells was monitored by FACS analysis. Figure 4 shows the incorporation of TiO_2_ NPs in transfected as well as un-transfected cells. When the cells were transfected with the TLR 4 expression vector, the uptake efficiency was increased approximately 2-fold compared with untransfected cells. In order to understand whether TiO_2_ required LBP and CD 14 for incorporation, cells were co-transfected with LBP:CD14:TLR 4 expression vectors. However, the FACS data did not show any variation in the uptake ratio between TLR 4 single transfected and LBP:CD 14:TLR 4 triple co-transfected cells. This suggested that TiO_2_ NPs did not undergo complex formation with LBP and CD 14 for incorporation into cells.

**Figure 5:**
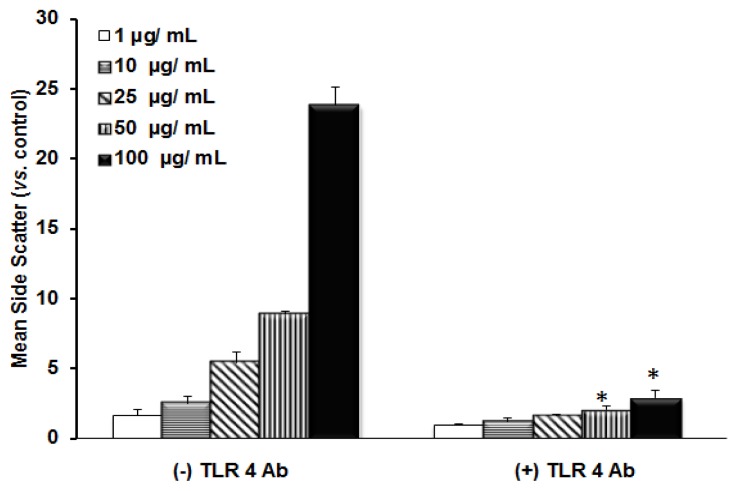
Inhibition in the incorporation of TiO_2_ NPs to NCI-H292 cells incubated with anti- TLR 4 Ab. NCI-H292 cells were incubated with anti-TLR 4 Ab followed by exposure to TiO_2_ NPs. The side scatter was analyzed and the mean side scatter for TiO_2_ NP-exposed cells was calculated from control cells (without NPs). The degree of significance for each concentration (1 μg/mL to 100 μg/mL) was calculated from the corresponding concentration of NP-exposed cells without TLR 4 Ab treatment. Each histogram represents each concentration of NPs. The data represent means ± SD, where *n* = 3. * *p* ≤ 0.005.

**Figure 6:**
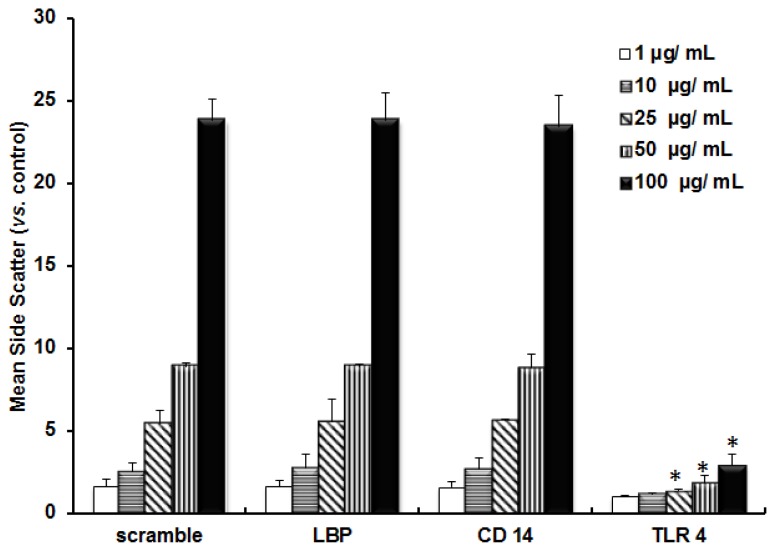
Difference in the incorporation of TiO_2_ NPs into NCI-H292 cells silenced with siRNAs. NCI-H292 cells were subjected to gene silencing with scrambled siRNA or siRNAs specific for LBP, CD 14, or TLR 4 followed by exposure to TiO_2_ NPs. Each histogram represents mean side scatter for each concentration of NPs. The data represent means ± SD, where *n* = 3. * *p* < 0.005 corresponds to scrambled siRNA.

The role of TLR 4 in the uptake of TiO_2_ NPs was also confirmed by anti-TLR 4 Ab treatment. Figure 5 shows the uptake of TiO_2_ NPs in the anti-TLR 4 Ab-treated cells as well as un-treated cells. The uptake ratio of TiO_2_ NPs was reduced when TLR 4 was blocked by anti-TLR 4 Ab. This indicated that TLR 4 was involved in the uptake of TiO_2_ NPs. The role of TLR 4 in the uptake of TiO_2_ NPs was also confirmed by gene knockdown experiments followed by exposure to TiO_2_ NPs (Figure 6) as mentioned previously. The amount of uptake of TiO_2_ NPs was significantly reduced in cells by TLR 4 knockdown, compared with scrambled siRNA. However, in the case of LBP and CD 14 knocked-down cells, there was no reduction in the uptake of TiO_2_ NPs. This suggested that TLR 4 is involved in the uptake of TiO_2_ NPs.

**Figure 7:**
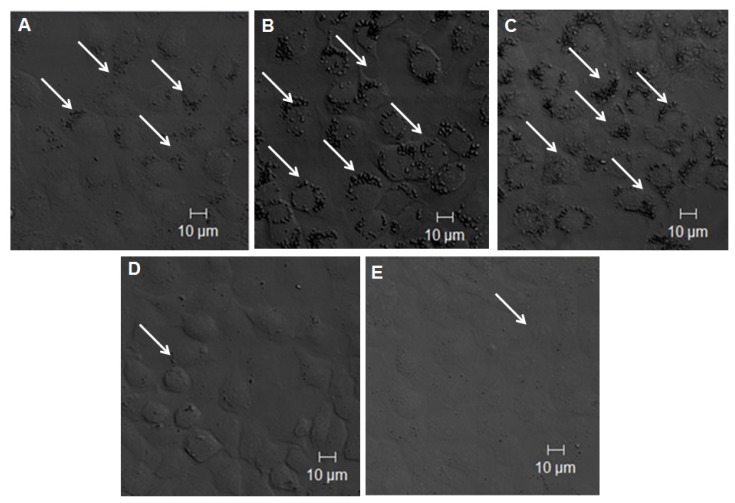
Confocal laser scanning microscopic images of TiO_2_ NP-exposed NCI-H292 cells. NCI-H292 cells without transfection (**A**) transfected with TLR 4 expression vector; (**B**) co-transfected with LBP, CD 14, and TLR 4 expression vectors; (**C**) with anti-TLR 4 Ab; (**D**) or TLR 4 siRNA; and (**E**) the confocal microscopic images show differences in the accumulation of TiO_2_ NPs inside the cells.

The incorporation of TiO_2_ NPs into cells was also confirmed by confocal microscopy. The cells were fixed and stained 24 h after exposure to NPs. Figure 7A–E shows the differences in the distribution of TiO_2_ NPs in the cells. The cells without transfection (Figure 7A) showed that TiO_2_ NPs were taken up and accumulated in the cytoplasm of the cells. Figure 7B shows the enhanced uptake of TiO_2_ NPs in the TLR 4-transfected cells. Figure 7C shows the results of cells co-transfected with LBP, CD 14, and TLR 4 expression vectors. The uptake of TiO_2_ NPs by these transfected cells was similar with that of TLR 4 expression vector single transfected cells. This also suggested that LBP and CD 14 were not involved in the uptake of TiO_2_ NPs. The induction of the uptake of TiO_2_ NPs by TLR 4 was also blocked by anti-TLR 4 Ab treatments (Figure 7D) as well as by TLR 4 gene knockdown experiments (Figure 7E). These results showed that down-regulation of TLR 4 expression reduced the uptake of TiO_2_ NPs. Confocal microscopic data also suggested that TLR 4, but not LBP or CD 14, is involved in the uptake of TiO_2_ NPs.

### 2.3. Discussion

A series of studies has shown that nanomaterials [34] such as silver NPs [35], carbon nanotubes [36], fullerenes [37], zinc oxide [38], and cerium oxide NPs [39] induce inflammatory responses. TiO_2_ NPs causes pulmonary inflammation [40], hepatocyte apoptosis [41], acute liver [42,43] and kidney injury [44], oxidative damage in lungs [45] and brain [46], nephrotoxicity [47] and reproductive system dysfunction [48] in mice. In our previous study, we showed that TiO_2_ NPs induce inflammatory markers such as IL-6 [32], and that PEG modification of TiO_2_ NPs reduces such inflammatory responses [33]. In the present study, we focused on the roles of LBP and CD 14 in uptake and inflammatory signaling via TLR 4 induced by TiO_2_ NPs.

TLRs play a fundamental role in the activation of innate immunity. TLRs have been studied for their role in the recognition of microbial pathogens. Each TLR recognizes a specific pathogen associated pattern. TLR 2, for example, forms heterodimers with TLR 1 or TLR 6 [49], and binds with several Gram-positive bacteria as well as bacterial cell wall components such as peptidoglycan and lipoteichoic acid (LTA). TLR 3 recognizes viral double stranded RNA [50]. TLR 4 binds with Gram-negative bacterial cell wall components such as LPS [51]. TLR 5 interacts with bacterial flagellin found in both Gram-positive and Gram-negative bacteria [52]. TLR 7 and TLR 8 detect viral infections [53]. TLR 9 acts as a receptor for bacterial unmethylated CpG DNA [54]. The exact function of TLR 10 is not known [55].

**Figure 8:**
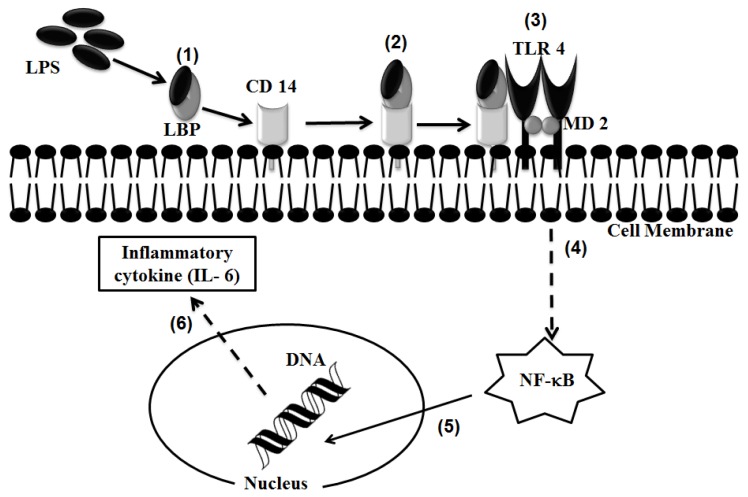
Schematic representation of the LPS-induced, TLR 4-mediated NF-κB signaling pathway: (1) LPS form Gram-negative bacteria complex with LPS-binding protein (LBP) in the cells; (2) This LPS and LBP complex then binds to CD 14 receptor; (3) The LPS, LBP, and CD 14 complexes in turn interact with MD 2 and the transmembrane protein TLR 4; (4) This induces the activation of the NF-κB signaling pathway; (5) Translocation of NF-κB to the nucleus induces the transcription of mRNA for several proteins such as inflammatory cytokines; (6) Translation of inflammatory cytokines such as IL-6.

In order to identify the role of TLR 4 in the TiO_2_ NP-induced inflammatory response, we analyzed the expression of IL-6 mRNA quantitatively by RT-PCR in a series of human expression vector transfected cells. Our findings showed that cells transfected with TLR 4, but not LBP or CD 14, had up-regulated inflammatory responses. This was also confirmed by blocking the function of TLR 4 with anti-TLR 4 Ab and in TLR 4 siRNA experiments. A previous study regarding the Ab assay showed that anti-TLR 4 Ab treatment decreases mucosal expression of CCL2, CCL20, TNF-α, and IL-6 [56]. Our data showed a reduction in the expression of IL-6 mRNA after TLR Ab treatment similar to TLR 4 siRNA experiments. The inflammatory agent LPS activates the secretion of proinflammatory agent TNF-α by binding with CD 14 [57]. The CD 14-dependent LPS uptake mechanism occurs in mCD 14 positive monocytes and endothelial cells [58]. To test whether LBP or CD 14 function in the TiO_2_ NP-mediated inflammatory response, we analyzed the expression of IL-6 mRNA in LBP and CD 14 knockdown cells. These results also confirmed that LBP and CD 14 are not involved in complex formation with TiO_2_ NPs. Figure 8 shows a schematic representation of the LPS-induced, TLR 4-mediated NF-κB signaling pathway. Here, our study focused on the roles of LBP and CD 14 in the uptake and signal transduction of TiO_2_ NPs via TLR 4.

To confirm the role of TLR 4 in the uptake of TiO_2_ NPs, we transfected cells with TLR 4 or LBP:CD 14:TLR 4 expression vectors and measured the uptake efficiency by FACS analysis. FACS data showed that TLR 4 is involved in the incorporation of TiO_2_ NPs into the cells, but LBP and CD 14 complexes are not. This was also confirmed by observing the cellular distribution of TiO_2_ NPs under confocal microscopy. Anti-TLR 4 Ab treatment and TLR 4 siRNA transfection reduced the uptake of TiO_2_ NPs. However, the existence of some TiO_2_ NPs in the cells indicated that these particles can be taken up by cells via endocytosis.

Our data show that TLR 4 is involved in the incorporation and inflammatory induction of TiO_2_ NPs; however, complex formation of LBP and CD 14 with TLR 4 was not necessary for the induction of the inflammatory response or for uptake into cells by TiO_2_ NPs. This suggested that TiO_2_ NPs can directly bind to TLR 4, similar to complexes of LPS, LBP, and CD 14. The results also suggested that character of TiO_2_ NPs might be similar to the complex of LPS, LBP and CD 14. These results are important for understanding the interactions between NPs and cells, which are in turn important for understanding the safety of using of NPs in medicine and other fields.

## 3. Experimental Section

### 3.1. Preparation of Titanium Dioxide Nanoparticles

The preparation of TiO_2_ NP aggregates was explained previously [32]. The initial particle size of TiO_2_ is 25 nm (Degussa Aeroxide P25) and has a crystal ratio of anatase 80% and rutile 20%. The average particle size and zeta potentials are 596 nm and −23.67 mV (Delsa™ Nano Particle Analyzer Beckman Coulter, Inc., Brea, CA, USA) respectively. Possible lipopolysaccharide (LPS) contamination in TiO_2_ NPs was checked by a ToxiSensor™ Chromogenic LAL Endotoxin Assay Kit (GenScript, Piscataway, NJ, USA) according to the manufacturer’s instructions. We could not detect any LPS contamination in NPs (data not shown).

### 3.2. Cell Culture

The human pulmonary epithelial cell line, NCI-H292 [59], was cultured in RPMI 1640 medium (Invitrogen, Grand Island, NY, USA) supplemented with 10% heat inactivated fetal bovine serum (FBS) (Biowest, Eckfield, East Sussex, UK), 100 U/mL penicillin, and 100 μg/mL streptomycin (Nacalai Tesque, Kyoto, Japan).The cells were maintained in a humidified atmosphere of 5% CO_2_ at 37 °C and were cultured in the dark to avoid the activation of the titanium surface.

### 3.3. Quantitative Real-Time (RT) PCR

For mRNA expression analysis, 1.3 × 10^5^ cm^−2^ of NCI-H292 cells were seeded in cell culture dishes (Corning Inc., Union City, NY, USA). Followed by overnight incubation, the cells were transfected with human expression vectors such as LBP (pUNO-hLBP) (InvivoGen, San Diego, CA, USA), CD 14 (pUNO-hCD14, InvivoGen), or TLR 4 (pUNO1-hTLR04a, InvivoGen) independently, as well as co-transfected with vectors such as LBP:TLR 4, CD14:TLR 4, or LBP:CD14:TLR 4 using Lipofectamine 2000 transfection reagent (Invitrogen, Carlsbad, CA, USA) according to the manufacturer’s instructions, and then incubated at 37 °C. The old medium was replaced 6 h post-transfection by fresh medium. TiO_2_ NP suspensions were prepared at a final concentration of 0.01 *w/v* % (100 μg/mL) and sonicated for 10 min using a bath-type sonicator (Ultrasonic cleaner, Iwaki, Japan) before being introduced into cells that were previously transfected with or without human expression vectors. The cellular responses of transfected cells and un-transfected cells were checked initially by exposure to LPS (Sigma-Aldrich, St. Louis, MO, USA) at a concentration of 20 ng/mL. Control cells were also maintained without LPS or NPs exposure. The fold of induction of interleukin 6 (IL-6) mRNA was measured as described in our previous papers [32,33], and the data were normalized to GAPDH.

Simultaneously, the transfected and untransfected cells were treated with human TLR 4 antibody (Ab) (MAb hTLR4, Invivogen) at a concentration of 1 μg/mL 1 h before exposure to LPS or TiO_2_ NPs. The relative fold of induction of IL-6 was analyzed by RT-PCR. The data were compared with that of cells without the addition of TLR 4 antibody.

To knockdown specific genes, the cells were also transfected with siRNAs specific for LBP, CD 14, or TLR 4 (Santa Cruz Biotechnology, Inc., Santa Cruz, CA, USA) according to the manufacturer’s instructions. 48 h post-transfection, the cells were treated with LPS or TiO_2_ NPs. Gene knockdown was confirmed by the analysis of endogenous expression of LBP, CD 14, or TLR 4 by RT-PCR using the following primers: LBP: forward primer, 5′-AAGGCCTGAGTCTCAGCATCTC-3′ and reverse primer, 5′-TGACTTGCGCACCTTCCA-3′; CD 14: forward primer, 5′-CGCTCCGAGATGCATGTG-3′ and reverse primer, 5′-AGCCCAGCGAACGACAGA-3′; TLR 4: forward primer, 5′-TTTTCCCTGGT GAGTGTGACTAT-3′ and reverse primer, 5′-TGAAGCAACTCTGGTGTGAGTA-3′. A scrambled siRNA (sc-36869, Santa Cruz Biotechnology, Inc., Santa Cruz, CA, USA) was used as a control in the same experiments.

### 3.4. Fluorescent Activated Cell Sorter (FACS) Analysis

For FACS, NCI-H292 cells were seeded at a concentration of 8 × 10^4^ cells/well in 24-well plates (Corning Inc., Union City, NY, USA). After overnight incubation, the cells were transfected and cotransfected with human expression vectors such as TLR 4 or LBP:CD 14:TLR4 as explained previously. The cells were also incubated with human TLR 4 antibody as mentioned previously at a concentration of 1 μg/mL for 1 h before exposure of TiO_2_ NPs for FACS analysis. Gene knockdown experiments were also performed for FACS analysis as mentioned in the previous section.

When the cells became 70%–80% confluent, they were treated with different concentrations of TiO_2_ suspensions ranging from 0.0001% (*w/v*) (1 μg/mL) to 0.01% (*w/v*) (100 μg/mL), and incubated for 24 h. After 24 h, the cells were collected by washing with phosphate buffered saline (PBS) followed by trypsinization and centrifugation, and resuspended in 1 mL PBS and placed on ice before analysis.

The amounts of particles taken up by the cells were analyzed using a FACS Calibur flow cytometer (Becton Dickinson, Franklin Lakes, NY, USA). About 10,000 events/sample were analyzed. The laser light scattered at narrow angles to the axis of the laser beam is called the forward-scatter (FSC), which is proportional to the size of the cells. Laser light scattered at about a 90° angle to the axis of the laser is called side-scatter (SSC), which is proportional to the intracellular density. The mean SSC for each concentration of NPs was calculated based on the peak intensities obtained for samples and controls (test sample/control) using Win-MDI 2.8 software (Joe Trotter, The Scripps Research Institute, La Jolla, CA, USA).

### 3.5. Confocal Laser Scanning Microscopic (CLSM) Observation

For confocal microscopy, 5 × 10^4^ cells/mL were seeded and incubated overnight. The cells were transfected with human TLR 4 expression vector or co-transfected with LBP:CD 14:TLR 4 as explained previously. TiO_2_ NP aggregates were applied at a concentration of 0.01 *w/v* % (100 μg/mL) to TLR 4 or LBP:CD 14:TLR 4 transfected cells. Simultaneously, the cells were treated with human TLR 4 antibody as explained previously. The cells were also subjected to TLR 4 siRNA transfection as mentioned before to knockdown the target genes.

The cells were fixed with 0.4% paraformaldehyde (PFA) after 24 h of exposure to the NPs. Microscopic images of fixed cells were obtained using CLSM (LSM510 META, Carl Zeiss, Germany).

### 3.6. Statistical Analysis

The data are expressed as means ± standard deviation (SD), where *n* ≥ 3. Analysis of data distribution was performed by Student’s *t*-test to analyze the significance of difference between the treated groups and control groups without NPs, human TLR 4 or LBP:CD 14:TLR 4 expression vector-transfected and un-transfected groups, TLR 4 antibody treated and untreated groups, and test siRNA transfected and scramble siRNA transfected groups.

## 4. Conclusions

We focused on the role of LBP and CD 14 in uptake and inflammatory signaling via TLR 4 induced by TiO_2_ NPs. Our findings showed that TLR 4 actively takes part in the uptake of TiO_2_ as well as inflammatory signal transduction, but LBP and CD 14 do not. TLR 4-overexpressing cells increased the uptake of TiO_2_ NPs and induced the up-regulation of inflammatory marker IL-6 mRNA, but LBP and CD 14 over-expressing cells did not. Our findings suggest that LPS binding receptors such as LBP and CD 14 do not take part in TLR 4-mediated NP uptake and signal transduction.
